# Nutritional compositions of Indian *Moringa oleifera* seed and antioxidant activity of its polypeptides

**DOI:** 10.1002/fsn3.1015

**Published:** 2019-04-02

**Authors:** Lili Liang, Cong Wang, Shaoguang Li, Xuemei Chu, Kunlai Sun

**Affiliations:** ^1^ Zhejiang Provincial Engineering Technology Research Center of Marine Biomedical Products, School of Food and Pharmacy Zhejiang Ocean University Zhoushan China; ^2^ Guangxi Key Laboratory of Chemistry and Engineering of Forest Products, Key Laboratory of Guangxi Colleges and Universities for Food Safety and Pharmaceutical Analytical Chemistry, School of Chemistry and Chemical Engineering Guangxi University for Nationalities Nanning China

**Keywords:** antioxidant activity, enzymatic hydrolysis, free radical scavenging activity, Indian* Moringa oleifera* seed, nutritional composition analysis

## Abstract

To study the nutritional composition of Indian *Moringa oleifera* seed and the antioxidant activity of *M. oleifera* seed polypeptide, Indian *M. oleifera* seed was used as raw material for composition analysis and content determination. After extraction of the seed protein, enzymatic hydrolysis with flavourzyme, dispase, papain, pepsin, and alcalase was conducted for different time, and the optimal enzymatic hydrolysis conditions was determined with DPPH scavenging capacity as an indicator. The seed polypeptides obtained by enzymatic hydrolysis were ultrafiltered, and the active peptide fragments were tracked with DPPH, HO (•OH), ABTS and superoxide anion (O_2_•^−^) free radical scavenging ability and lipid oxidation inhibition rate as indicators. The results showed that the protein content in Indian *M. oleifera* seed was high to 40.34%, containing seven essential amino acids. The content of macroelements such as potassium, sodium, and magnesium is high, with the potassium content as high as 2,357.71 mg/kg, among the microelements, the iron content as high as 36.2 mg/kg. The optimum enzymatic hydrolysis conditions were as follows: enzymatic hydrolysis with flavourzyme (50°C, pH 6.7) for 300 min, and DPPH scavenging capacity was 84.76%. Activity tracing found that the polypeptide fragment with molecular weight <3.5 kDa had the strongest antioxidant capacity, and the EC_50_ values of DPPH, •OH, ABTS, and O_2_•^−^ free radical scavenging rates were 4.0, 4.2, 5.3, and 4.3 mg/ml, respectively. The above results show that Indian *M. oleifera* seed not only has high nutritional value, but its protease enzymatic hydrolyzate also has significant antioxidant activity, which can be further developed into nutrition products, healthcare products, functional foods, beauty and skin care products, liver protection drugs, etc.

## 1 INTRODUCTION

*Moringa oleifera* lam is a perennial woody plant of *Moringa* family, originating in arid or semiarid regions of tropical and southern subtropics (Yiftach & Adina, 2017). At present, more than 30 countries in the world have introduced and cultivated *Moringa*. Guangdong, Guangxi, Hainan, Sichuan, and Yunnan provinces of China have introduced *M. oleifera* seeds or cultivation techniques from countries such as India and Myanmar. *M. oleifera* seed is rich in oils, proteins, and minerals. It can be used in food, medicine, cosmetics, and water purification, and has good research prospects. It has significant effects in reducing blood lipids, blood pressure, slimming, regulating the stomach, protecting the liver from alcohol, and enhancing the body's immunity (Du, Sun, Yan, Luo, & Dai, 2017; González et al., 2017; Pereira, Oliverira, & Oliverira, 2011). In addition, studies have confirmed that *M. oleifera* protein is soluble in aqueous solution and can be completely digested (Fan, Shao, Ye, & Yang, 2016). Lin found that *M. oleifera* seed polypeptide has protective effect on the erythrocytes which suffered oxidative damage (Lin, Zhu, & Zhao, 2018), and Zhao found the extract of *M. oleifera* leaves can strongly scavenge DPPH and superoxide anion free radical and absorb oxidative free radical (Zhao, Li, Lin, & Yang, 2017). *M. oleifera* root protein has anti‐inflammatory and analgesic functions. At present, there are few studies on *M. oleifera* seed protein. The only articles are the research provided by Aderinola and his coworkers. Their study showed *M. oleifera *seed protein has antioxidant properties (Aderinola, Fagbemi, Enujiugha, Alashi, & Aluko, 2018), especially after hydrolysis by trypsin or alcalase, the hydrolyzate shows in vitro antihypertensive and antioxidative properties (Aderinola, Fagbemi, Enujiugha, Alashi, & Aluko, 2019a, 2019b). In this article, we analyze the Indian *M. oleifera* seed nutrient composition and the antioxidant activity of its peptides hydrolyzed by flavourzyme, dispase, papain, pepsin, and alcalase, expected to provide data support for the in‐depth research and development of *M. oleifera* seed polypeptide.

## 2 MATERIALS AND METHODS

### 2.1 Materials and reagents

*Moringa oleifera* seeds were collected in the southern Himalayas of Assam, North India (94.1°E, 26.7°N).

DPPH: Shanghai Macklin Biochemical Co., Ltd; FeSO4 (AR): Wuxi Jingke Chemical Co., Ltd.; ABTS: Beijing Biotopped Science & Technology Co., Ltd.; NBT, NADH: Thermo Fisher Scientific; PMS: Aladdin; pepsin, flavourzyme, alcalase: Beijing Solarbio Science & Technology Co., Ltd.; dispase, papain: Novozymes Biotechnology Co., Ltd.; anhydrous ethanol, phenanthroline, sodium dihydrogen phosphate, disodium hydrogen phosphate, potassium persulfate, trichloroethanol, thiobarbituric acid, ascorbic acid, etc. were all analytical reagents provided by Sinopharm Chemical Reagent Co., Ltd.

### 2.2 Instrument

BSA224 Analytical Balance: Beijing Aoduolisi Scientific Instrument Co., Ltd.; LGJ‐10C ordinary vacuum freeze dryer: Four‐ring Science Instrument Plant Beijing Co., Ltd.; HH‐8 homoeothermic water bath: Jintan Sita Xinbao Instrument Factory; UV‐1100 UV spectrophotometer: Shanghai Mapada Instruments Co., Ltd.; WTM‐1812G membrane separation equipment: Hangzhou Woteng Membrane Engineering Co., Ltd.; MK3 microplate reader: Thermo Fisher Scientific Co., Ltd.; N‐1100 Rotary Evaporator: Shanghai Ailang Instrument Co., Ltd.; STARTER3100 Precision pH Meter: Shanghai Ohaus Instrument Co., Ltd.; SH8210HP ultrasonic cleaning agent: Shanghai Kudos Ultrasonic instrument Co., Ltd.; JJ‐02 high speed pulverizer: Jiangsu Changzhou Huaguo Electric Appliance Co., Ltd.; TGL‐16M high speed centrifuge: Shanghai Lu Xiangyi Centrifuge Instrument Co., Ltd.; L‐8900 amino acid analysis: Techcomp (China) Scientific Instrument Co., Ltd.; ATN‐300 Automatic Kjeldahl Nitrogen Analyzer: Shanghai Hongji Instrument Co., Ltd.; KSW‐4D‐11‐S resistance furnace temperature controller: Shanghai Xian Jian Instrument Co., Ltd.

### 2.3 Experimental methods

#### 2.3.1 Determination of nutrients

##### Determination of general nutrients

Determination of crude protein content refers to GB/T5009.5‐2016 “Determination of protein in food,” using Kjeldahl method; determination of ash content refers to GB 5009.4‐2016 “Determination of ash in food,” using muffle furnace burning method; determination of crude fat refers to GB 5009.6‐20163 “Determination of fat in food,” using Soxhlet extraction method; determination of mineral elements refers to GB5009.268‐2016 “Determination of multi‐elements in food”; determination of moisture refers to GB5009.3‐2016 “Determination of moisture in food,” using direct drying method; determination of nucleic acid refers to GB5009.87‐2016 “Determination of phosphorus in food,” using Fiske‐Subbarow method; and determination of carbohydrate acid refers to NY/T 2332‐2013.

##### Determination of amino acid composition and content

After the *M. oleifera* seeds were dried and pulverized, 0.5 g was weighed and was digested with 6 mol/L hydrochloric acid at a constant temperature of 110°C for 22 hr, and then the amino acid content was determined by an L‐8900 amino acid analyzer (Hwee & Chee, 2013; Zhang, 2016).

#### 2.3.2 Method for extracting crude protein from *M. oleifera* seed

Smash, pass 80‐mesh sieve → ethyl acetate degreasing (ratio of liquid to material 1:6 mg/ml, 48 hr)→air‐drying (37°C, 48 hr)→ addition of Tris‐HCL protein extraction with 1.5 mol/L concentration and 8.8 pH (ratio of liquid to material 1:38 mg/ml, 42°C, 100 min)→obtain supernatant after centrifugalization (2,810 *g*, 15 min)→ammonium sulfate precipitation (4.25 mol/L, 25°C)→ dialysis desalting (10 kDa, 25°C, 48 hr)→ freeze‐drying (−60°C, 36 hr).

#### 2.3.3 Determination of the antioxidant activity of *M. oleifera* seed protein, Vc as positive control

##### Determination of the scavenging ability of *M. oleifera* seed protein to DPPH free radicals

Two milliliters of samples consisting of distilled water and different concentrations of the analytes were placed in cuvettes, and 500 μl of an ethanolic solution of DPPH (0.02%) and 1.0 ml of ethanol were added. A control sample containing the DPPH solution without the sample was also prepared. In the blank, the DPPH solution was substituted with ethanol. The antioxidant activity of the sample was evaluated using the inhibition percentage of the DPPH radical with the following equation:

$$\text{DPPH free radical scavenging rate}=\frac{A_{\text{c}}+A_{\text{o}}-A_{\text{s}}}{A_{\text{c}}}\times 100\%.$$

*A*_c_: Absorbance value of the control group; *A*_o_: Absorbance value of the blank group; *A*_s_: Absorbance value of the sample group (Pan, Zhao, Hu, & Wang, 2016).

##### Determination of the scavenging ability of *M. oleifera* seed protein to hydroxyl radicals

1.0 ml of a 1.87 mM 1,10‐phenanthroline solution and 2.0 ml of the sample were added to a screw‐capped tube and mixed. Then, 1.0 ml of a FeSO_4_·7H_2_O solution (1.87 mM) was added to the mixture. The reaction was initiated by adding 1.0 ml of H_2_O_2_ (0.03%, v/v). After incubating at 37°C for 60 min in a water bath, the absorbance of the reaction mixture was measured at 536 nm against a reagent blank. The reaction mixture without any antioxidant was used as the negative control, and a mixture without H_2_O_2_ was used as the blank. The hydroxyl radical scavenging activity (HRSA) was calculated using the following formula:

$$\text{HRSA}\;\left(\%\right)=\frac{A_{\text{s}}-A_{\text{c}}}{A_{\text{o}}-A_{\text{c}}}\times 100\%.$$

*A*_c_: Absorbance value of the control group; *A*_o_: Absorbance value of the blank group; *A*_s_: Absorbance value of the sample group (Wang et al., 2014).

##### Determination of the scavenging ability of *M. oleifera* seed protein to ABTS free radical

ABTS^•+^ was generated by mixing an ABTS stock solution (7 mM) with potassium persulfate (2.45 mM). The mixture was left in the dark at room temperature for 16 hr. The ABTS radical cation solution was diluted in 5 mM phosphate‐buffered saline (PBS) pH 7.4 to an absorbance of 0.70 ± 0.02 at 734 nm. One milliliter of diluted ABTS^•+^ solution was mixed with one milliliter of the different sample concentrations. Ten minutes later, the absorbances were measured at 734 nm against the corresponding blank. The ABTS^•+^ scavenging activities of the samples were calculated using the following equation:

$$\text{ABTS free radical scavenging rate}\;=\frac{A_{\text{c}}-A_{\text{s}}}{A_{\text{c}}}\times 100\%.$$

*A*_c_: Absorbance value of the control group; *A*_s_: Absorbance value of the sample group.

##### Determination of the scavenging ability of *M. oleifera* seed protein to superoxide anion

In the experiment, superoxide anions were generated in 1 ml of nitrotetrazolium blue chloride (NBT) (2.52 mM), 1 ml of NADH (624 mM), and 1 ml of different sample concentrations. The reaction was initiated by adding 1 ml of phenazine methosulfate (PMS) solution (120 µM) to the reaction mixture. The absorbance was measured at 560 nm against the corresponding blank after 5‐min incubation at 25°C. The scavenging capacity of the O_2_^−• ^was calculated using the following equation:

$$\text{Superoxide anion scavenging rate}\;=\frac{A_{\text{c}}-A_{\text{s}}}{A_{\text{s}}}\times 100\%.$$

*A*_c_: Absorbance value of the control group; *A*_s_: Absorbance value of the sample group.

##### Determination of antilipid peroxidation capacity of *M. oleifera* seed protein

Briefly, a sample (5.0 mg) was dissolved in 10 ml of 50 mM PBS (pH 7.0) and added to 0.13 ml of a solution of linoleic acid and 10 ml of 99.5% ethanol. Then, the total volume was adjusted to 25 ml with deionized water. The mixture was incubated in a conical flask with a screw cap at 40°C in a dark room, and the degree of oxidation was evaluated by measuring ferric thiocyanate values. The reaction solution (100 μl) incubated in the linoleic acid model system was mixed with 4.7 ml of 75% ethanol, 0.1 ml of 30% ammonium thiocyanate, and 0.1 ml of 20 mM ferrous chloride solution in 3.5% HCl. After 3 min, the thiocyanate value was measured at 500 nm following color development with FeCl_2_ and thiocyanate at different intervals during the incubation period at 40°C (Sudhakar & Nazeer, 2015).

#### 2.3.4 Polypeptide separation and purification

##### Enzymatic hydrolysis

The protein solution with concentration of 0.01 g/ml and the enzyme with concentration of 0.005 g/ml were extracted from *M. oleifera* seed, and the enzymatic hydrolysis time was set to 8 time gradients of 30, 60, 120, 180, 240, 300, 360, and 420 min. Enzymatic hydrolysis with flavourzyme (50°C, pH 6.7; Ji, 2017), dispase (55°C, pH 7; Zhong, Chen, & Wen, 2009), papain (50°C, pH 7; Jiang, Wu, Wang, & Zhang, 2014), pepsin (32°C, pH 2.0; Feng, Ruan, Jin, Xu, & Wang, 2018), and alcalase (55°C, pH 10; Yang, Fan, Shao, & Wang, 2015) was conducted, respectively, and DPPH scavenging capacity was used as an indicator to select the optimal enzymatic hydrolysis conditions. The DPPH scavenging capacity is determined as above.

##### Ultrafiltration

Peptide fragments with different molecular sizes of >5 kDa, 3.5–5 kDa, and <3.5 kDa were obtained after ultrafiltration using a 5 kDa, 3.5 kDa ultrafiltration membrane. The three sections of the solution were separately collected for lyophilization, and the dried peptide was stored at −20°C.

##### Screening for active components

The ability of the peptide fragments with three different molecular sizes to scavenge DPPH, •OH, ABTS, O_2_•^− ^free radicals and the EC_50_ values of lipid antioxidants were studied to screen for more active components. The DPPH•, •OH, ABTS•, O_2_•^− ^scavenging power and lipid antioxidants were determined as above.

## 3 EXPERIMENTAL RESULTS

### 3.1 Proximate composition of *M. oleifera* seed

As can be seen from Table 1, the content of crude protein and crude lipid in Indian *M. oleifera* seed is relatively rich, which are 40.34% and 39.12%, respectively. These are very close to those reported in the literature; however, protein is much more than that in *M. oleifera* leaves (28.73%) (Juhaimi, Ghafoor, Ahmed, Babiker, & Ozcan, 2017). Compared the content of crude protein and lipid with the well‐recognized peanuts (32.65% and 48.06%) and soybean (41.05% and 21.06%) (Liu & Pan, 2016; Zheng, Jin, Geng, & Yu, 2015), the crude protein content of Indian *M. oleifera* seed is equivalent to soybean, higher than peanut; its crude lipid content is much higher than soybeans, slightly lower than peanuts. It is reported that *M. oleifera* seed protein has the effect of water purification and anticoagulation (Aline et al., 2017; Luciana et al., 2013; Marichamy & Ramasamy, 2015), and *M. oleifera* oil can accelerate the healing of wounds (Duan et al., 2011). It shows that the Indian *M. oleifera* seed is not only rich in crude protein and lipid, but also has various effects. We can deeply develop its plant protein and vegetable oil, such as functional foods, feed, water purification, cosmetic raw materials, plant growth promoters, and fungicides.

**Table 1:** General nutrient content of Indian *Moringa oleifera* seed (%), *p* ≤ 0.05

Crude protein	Ash	Water	Crude lipid	Carbohydrate	Nucleic acid
40.34 ± 0.45	3.5 ± 0.31	6.78 ± 0.61	39.12 ± 0.42	8.94 ± 0.28	4.26 ± 0.37

### 3.2 Content of mineral elements in *M. oleifera* seed

As can be seen from Table 2, *M. oleifera* seed contains potassium, sodium, magnesium, calcium, and other elements, of which the highest potassium content is 2,357.71 mg/kg and the lowest calcium content is 121.14 mg/kg. The contents of the four macroelements are K > Na > Mg > Ca in order, which is different in order and much less than those contains in leaves (K, Mg, Ca: 3,562.19, 3,813.44, 17,638.41 mg/kg, respectively). At the same time, microelements such as iron, copper, and zinc were detected. The highest iron content is 36.2 mg/kg. The contents of three microelements are Fe > Zn > Cu in order, which is also different in order and much less than those contains in leaves (Fe, Zn, Cu: 1,274.12, 29.51, 8.41 mg/kg, respectively) (Juhaimi, Ghafoor, Babiker, Matthaus, & Ozcan, 2017). *M. oleifera* seeds also contain four heavy metal elements of arsenic, cadmium, lead, and tin, which are 0.01, 0.018, 0.015, and 0.007 mg/kg, respectively. These are low in content and are in line with GB2762‐2017 “The national food safety standard, food contaminants limit” (arsenic is limited to 0.5 mg/kg in grains and their products; cadmium and lead are limited to 0.5 and 0.2 mg/kg, respectively; and in nuts and seeds, tin is limited to 250 mg/kg in food).

**Table 2:** Content of mineral elements in *Moringa oleifera* seed (mg/kg DW, *p* ≤ 0.05)

Mineral elements	Contents	Mineral elements	Contents
K	2,357.71 ± 1.87	Cu	3.29 ± 0.21
Na	1,074.09 ± 1.56	As	0.01 ± 0.001
Mg	972.06 ± 1.23	Cd	0.018 ± 0.002
Ca	121.14 ± 0.87	Pb	0.015 ± 0.001
Fe	36.20 ± 0.79	Sn	0.007 ± 0.001
Zn	8.37 ± 0.12		

### 3.3 Amino acid composition and content in *M. oleifera* seeds

As can be seen from Table 3, *M. oleifera* seed contains seven essential amino acids, namely threonine, valine, methionine, isoleucine, leucine, phenylalanine, and lysine, with the total content of 0.824 g/100g, accounting for 25.65% of the total amino acid content. This result is close to the 28.8% of essential amino acids in Liaoning peanuts reported by Shi (Shi, Yu, & Han, 2017). The *M. oleifera* seeds contain seven kinds of hydrophobic amino acids: alanine, valine, methionine, isoleucine, leucine, tyrosine, and phenylalanine, and the total content is 0.834 g/100g. The antioxidant activity of the protein is closely related to its amino acid composition. Peptides containing hydrophobic amino acids can increase their solubility at the water–lipid interface and better interact with free radicals (Zheng, Si, Baseer, Li, & Zhang, 2018).

**Table 3:** Amino acid composition and content analysis of *Moringa oleifera* seed, *p* ≤ 0.05

Amino acid	Contents	Amino acid	Contents
Aspartic acid (Asp)	0.164 ± 0.002	Isoleucine (lle)	0.087 ± 0.001
Threonine (Thr)	0.095 ± 0.004	Leucine (Leu)	0.214 ± 0.007
Serine (Ser)	0.130 ± 0.008	Tyrosine (Tyr)	0.018 ± 0.006
Glutamate (Glu)	0.839 ± 0.009	Phenylalanine (Phe)	0.165 ± 0.004
Proline (Pro)	0.196 ± 0.003	Lysine (Lys)	0.073 ± 0.009
Glycine (Gly)	0.200 ± 0.012	Histidine (His)	0.098 ± 0.006
Alanine (Ala)	0.160 ± 0.009	Arginine (Arg)	0.546 ± 0.009
Cysteine (Cys)	0.040 ± 0.005	Essential amino acid	0.82 ± 0.07
Valine (Val)	0.125 ± 0.008	Total amount of amino acids	3.22 ± 0.12
Methionine (Met)	0.065 ± 0.009		

### 3.4 Protein antioxidant activity

**Figure 1:**
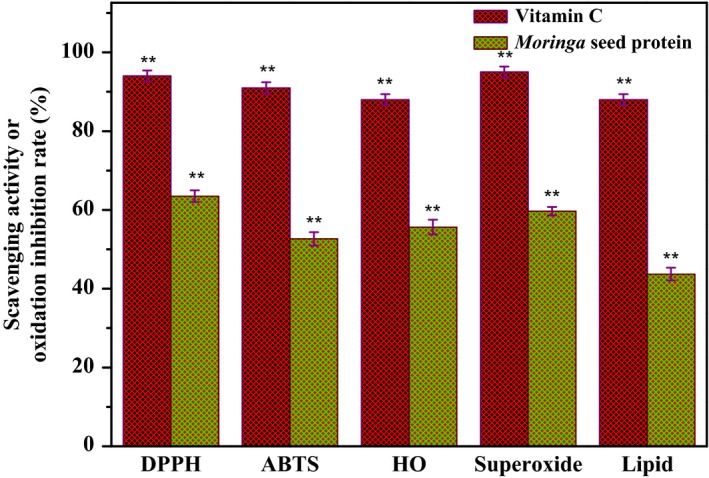
DPPH, •OH, ABTS, and O_2_•^−^ scavenging rate and lipid oxidation inhibition rate when the *Moringa oleifera* seed protein is 10 mg/ml

As shown in Figure 1, when the *M. oleifera* seed protein was 10 mg/ml, the DPPH•, ABTS•, •OH, and O_2_•^− ^scavenging rates were 63.25%, 52.45%, 55.25%, and 59.32%, respectively. And the lipid oxidation inhibition rate was 43.25%. It shows that *M. oleifera* seed protein extraction has good antioxidant activity against different antioxidant models.

### 3.5 Optimal enzymatic hydrolysis conditions

**Figure 2:**
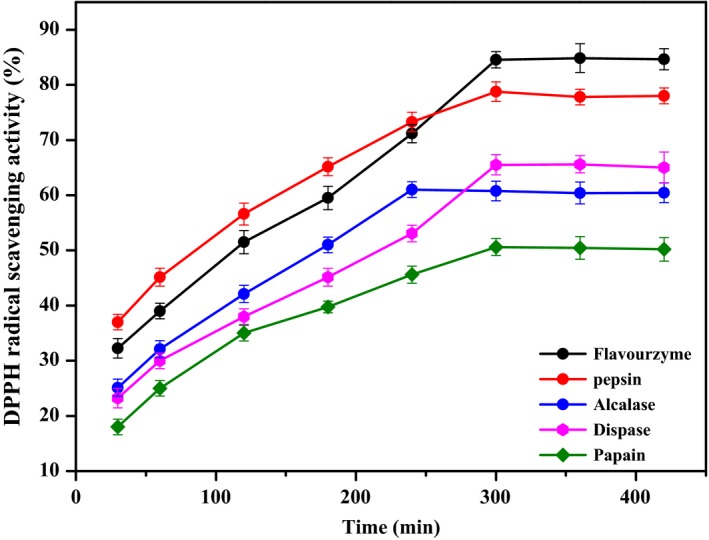
DPPH scavenging rate of enzymatic hydrolyzates using five kinds of protease at different enzymatic times

Flavourzyme (50°C, pH 6.7), dispase (55°C, pH 7.0), papain (50°C, pH 7.0), pepsin (32°C, pH 2.0), and alcalase (55°C, pH 10.0) were used to conduct enzymatic hydrolysis of *M. oleifera* seed protein for 30, 60, 120, 180, 240, 300, 360, and 420 min, and then the DPPH scavenging rate was determined. The results are shown in Figure 2.

As shown in Figure 2, the DPPH scavenging rate of the five enzymatic hydrolyzates increased gradually with the increase in enzymatic time. After 300 min, the scavenging rate tended to be stable, and the enzymatic hydrolysis was completed at this time. When the flavourzyme was used for the enzymatic hydrolysis of the *M. oleifera* seed protein for 300 min at 50°C and pH 6.7, the DPPH scavenging rate was up to 85%. Therefore, this condition was selected as the optimum enzymatic hydrolysis process for *M. oleifera* seed protein for subsequent studies.

### 3.6 Antioxidant activity of different peptide fragments

**Figure 3:**
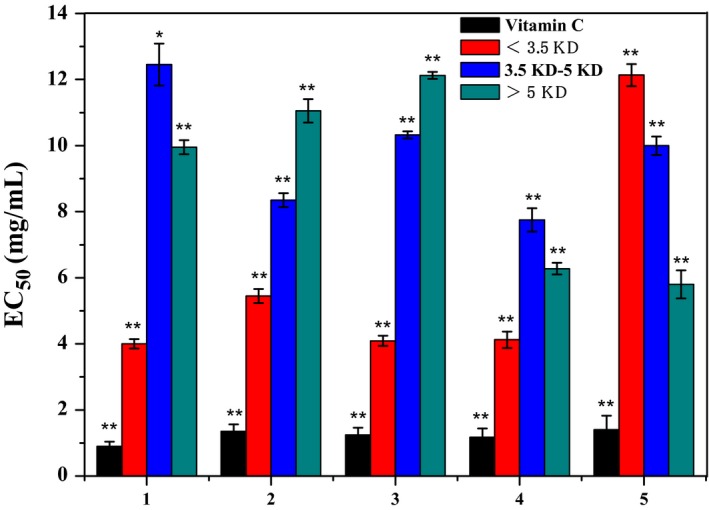
EC_50_ values of DPPH, •OH, ABTS, and O_2_•^−^ radical scavenging rate and lipid oxidation inhibition rates of peptide fragments with different molecular weights

After ultrafiltration, the enzymatic peptide was divided into three parts, the molecular weight of the peptide was <3.5 kDa, 3.5–5 kDa, >5 kDa, and the EC_50_ values of DPPH, •OH, ABTS, O_2_•^− ^free radical scavenging rate and the lipid antioxidant inhibition rate are shown in Figure 3.

As shown in Figure 3, for the peptide fragment with molecular weight <3.5 kDa, the EC_50_ values of DPPH, •OH, ABTS, and O_2_•^−^ scavenging rates were 4.0, 4.2, 5.3, and 4.3 mg/ml, respectively, and were less than the EC_50_ values of the peptide fragments of 3.5–5 kDa and >5 kDa, which indicates that their antioxidant activity is superior to the latter. The activity of the lipid antioxidant inhibition rate was a component having a molecular weight of >5 kDa and an EC_50_ value of 5.8 mg/ml. In summary, peptide fragment with the molecular weight <3.5 kDa has the strongest antioxidant capacity.

## 4 CONCLUSIONS

Indian *M. oleifera* seeds are rich in crude protein and crude lipids, with a protein content up to 40.34% and a crude lipids content 39.12%. Its content of macroelements such as sodium, potassium, and magnesium is much higher than other metals, and the K content is 2,537.71 mg/kg. Indian *M. oleifera* seeds contain seven essential amino acids and seven hydrophobic amino acids, and the hydrophobic amino acids contribute to the antioxidant activity of *M. oleifera* seeds. The optimum enzymatic conditions were flavourzyme hydrolysis of *M. oleifera* seed protein for 300 min at 50°C and pH 6.7. <3.5 kDa polypeptide fragment has higher EC_50_ values for DPPH•, •OH, ABTS•, and O_2_•^− ^scavenging rates than fragments 3.5–5 kDa and >5 kDa, and only the lipid oxidation inhibition rate of <3.5 kDa polypeptide fragment is lower than >5 kDa. Therefore, the <3.5 kDa polypeptide fragment is the strongest antioxidant. It has the potential to be further developed into nutritional products, health products, functional foods, beauty and skin care products, and liver protection drugs.

## CONFLICTS OF INTEREST

The authors declare no conflict of interest.

## ETHICS STATEMENT

This study has nothing to do with human and animal testing.

## References

[fsn31015-bib-0001] Aderinola, T. A. , Fagbemi, T. N. , Enujiugha, V. N. , Alashi, A. M. , & Aluko, R. E. (2018). Amino acid composition and antioxidant properties of *Moringa oleifera* seed protein isolate and enzymatic hydrolysates. Heliyon, 4, e00877 10.1016/j.heliyon.2018.e00877 30386828 PMC6205298

[fsn31015-bib-0002] Aderinola, T. A. , Fagbemi, T. N. , Enujiugha, V. N. , Alashi, A. M. , & Aluko, R. E. (2019a). In vitro antihypertensive and antioxidative properties of alcalase‐derived *Moringa oleifera* seed globulin hydrolysate and its membrane fractions. Journal of Food Processing and Preservation, 43, e13862 10.1111/jfpp.13862 PMC6341156 30680166

[fsn31015-bib-0003] Aderinola, T. A. , Fagbemi, T. N. , Enujiugha, V. N. , Alashi, A. M. , & Aluko, R. E. (2019b). In vitro antihypertensive and antioxidative properties of trypsin‐derived *Moringa oleifera* seed globulin hydrolyzate and its membrane fractions. Food Science and Nutrition, 7, 132–138. 10.1002/fsn3.826 30680166 PMC6341156

[fsn31015-bib-0004] Aline, T. A. B. , Mariana, O. S. , Raquel, G. G. , Rosângela, B. , Marcelo, F. V. , & Angélica, M. S. V. (2017). Protein fractionation of seeds of Moringa oleifera lam and its application in superficial water treatment. Separation and Purification Technology, 180, 114–124. 10.1016/j.seppur.2017.02.040

[fsn31015-bib-0005] Du, T. T. , Sun, P. , Yan, Q. X. , Luo, X. L. , & Dai, H. (2017). A comprehensive study of the Moringa seed. Popular Science & Technology, 19(12), 46–49.

[fsn31015-bib-0006] Duan, Q. F. , Li, Q. , Lin, Q. , Duan, X. H. , Yang, L. , Zhang, Z. Q. , … Wang, Y. Q. (2011). Protection effect of Moringa oil on skin injury of rabbit. Natural Product Research and Development, 23, 159–162. 10.16333/j.1001-6880

[fsn31015-bib-0007] Fan, J. L. , Shao, J. L. , Ye, Y. P. , & Yang, D. S. (2016). Determination on nutritional components in seeds of *Moringa oleifera* . Food and Nutrition in China, 22(5), 69–72.

[fsn31015-bib-0008] Feng, Y. X. , Ruan, G. R. , Jin, F. , Xu, J. , & Wang, F. J. (2018). Purification, identification, and synthesis of five novel antioxidant peptides from Chinese chestnut (*Castanea mollissima* Blume) protein hydrolysates. LWT‐ Food Science and Technology, 92, 40–46. 10.1016/j.lwt.2018.01.006

[fsn31015-bib-0009] González, G. N. G. , Chuc, K. J. A. , Torres, C. J. A. , Zambrano, E. A. G. , Ancona, D. B. , Guerrero, L. C. , & García, S. R. S. (2017). Biofunctional properties of bioactive peptide fractions from protein isolates of *moringa *seed (*Moringa oleifera*). Journal of Food Science and Technology, 54(13), 4268–4276. 10.1007/s13197-017-2898-8 29184233 PMC5686007

[fsn31015-bib-0010] Hwee, L. S. , & Chee, Y. G. (2013). Extraction of antioxidative and antihypertensive bioactive peptides from *Parkia speciosa* seeds. Food Chemistry, 141(4), 3435–3442. 10.1016/j.foodchem.2013.06.030 23993504

[fsn31015-bib-0011] Ji, S. H. (2017). Study on isolation and purification process of silkworm chrysalis polypeptide. Food Industry, 38(06), 101–103.

[fsn31015-bib-0012] Jiang, T. L. , Wu, H. Y. , Wang, W. , & Zhang, Z. Q. (2014). Optimization of antibacterial peptides preparation using pepsin from prickly ash seed protein by response surface methodology. Science and Technology of Food Industry, 35(20), 226–231. 10.13386/j.issn1002-0306.2014.20.041

[fsn31015-bib-0013] Juhaimi, F. A. , Ghafoor, K. , Ahmed, I. A. M. , Babiker, E. E. , & Ozcan, M. M. (2017). Comparative study of mineral and oxidative status of *Sonchus oleraceus*, *Moringa oleifera* and *Moringa peregrina* leaves. Journal of Food Measurement and Characterization, 11(4), 1745–1751. 10.1007/s11694-017-9555-9

[fsn31015-bib-0014] Juhaimi, F. A. , Ghafoor, K. , Babiker, E. E. , Matthaus, B. , & Ozcan, M. M. (2017). The biochemical composition of the leaves and seeds meals of *Moringa* species as non‐conventional sources of nutrients. Journal of Food Biochemistry, 41(1), e12322 10.1111/jfbc.12322

[fsn31015-bib-0015] Lin, L. Z. , Zhu, Q. Y. , & Zhao, M. M. (2018). Preparation of antioxidant peptides from *Moringa oleifera *seed and their protective effects on oxidatively damaged erythrocytes. Food Science, 8(25), 1754–9.

[fsn31015-bib-0016] Liu, W. J. , & Pan, W. (2016). Analysis of peanut nutrition quality evaluation and grey correlation method of edible quality in Fujian Province. Journal of Anhui Agricultural Sciences, 44(22), 88–92. 10.13989/j.cnki.0517-6611.2016.22.029

[fsn31015-bib-0017] Luz, L. D. A. , Silva, M. C. C. , Ferreira, R. D. S. , Santana, L. A. , Silva‐Lucca, R. A. , Mentele, R. , … Coelho, L. C. B. B. (2013). Structural characterization of coagulant *Moringa oleifera* Lectin and its effect on hemostatic parameters. International Journal of Biological Macromolecules, 58, 31–36. 10.1016/j.ijbiomac.2013.03.044 23537800

[fsn31015-bib-0018] Marichamy, M. , & Ramasamy, K. (2015). Modelling the kinetics of coagulation process for tannery industry effluent treatment using *Moringa oleifera* seeds protein. Desalination and Water Treatment, 57(32), 1754–11. 10.1080/19443994.2015.1070294

[fsn31015-bib-0019] Pan, X. , Zhao, Y. Q. , Hu, F. Y. , & Wang, B. (2016). Preparation and identification of antioxidant peptides from protein hydrolysate of skate (*Raja porosa*) cartilage. Journal of Functional Foods, 25, 220–230. 10.1016/j.jff.2016.06.008

[fsn31015-bib-0020] Pereira, M. L. , Oliverira, H. D. , & Oliverira, J. T. A. (2011). Purification of a chitin‐binding protein from *Moringa oleifera* seeds with potential to relieve pain and inflammation. Protein and Peptide Letters, 18, 1078–1085. 10.1371/journal.pbio.1001191 21675945

[fsn31015-bib-0021] Shi, T. Y. , Yu, M. , & Han, Y. Q. (2017). The nutrients and characteristic analysis of Liaoning peanut varieties. Food Research and Development, 38(22), 142–147. 10.3969/j.issn.1005-6521.2017.22.029

[fsn31015-bib-0022] Sudhakar, S. , & Nazeer, R. A. (2015). Structural characterization of an Indian squid antioxidant peptide and its protective effect against cellular reactive oxygen species. Journal of Functional Foods, 14, 502–512. 10.1016/j.jff.2015.02.028

[fsn31015-bib-0023] Wang, Q. , Li, W. , He, Y. , Ren, D. , Kow, F. , Song, L. , & Yu, X. (2014). Novel antioxidative peptides from the protein hydrolysate of oysters (*Crassostrea talienwhanensis*). Food Chemistry, 145(7), 991–996. 10.1016/j.foodchem.2013.08.099 24128574

[fsn31015-bib-0024] Yang, D. S. , Fan, J. L. , Shao, J. L. , & Wang, L. X. (2015). Comparative analysis of nutritional components and amino acid composition of different parts of *Moringa oleifera* Lam. Journal of Shanxi Agricultural Sciences, 43(9), 1110–1115. 10.3969/j.issn.1002-2481.2015.09.11

[fsn31015-bib-0025] Yiftach, V. , & Adina, M. (2017). The potential of the tropical “miracle tree” *Moringa oleifera* and its desert relative *Moringa* peregrina as edible seed‐oil and protein crops under Mediterranean conditions. Scientia Horticulturae, 225, 431–437. 10.1016/j.scienta.2017.07.039

[fsn31015-bib-0026] Zhang, H. (2016). Study on bioactive ingredients of *Panax* ginseng C. A. Meyer for protein, amino acids, organic acids and nucleosides [D]. Jilin University.

[fsn31015-bib-0027] Zhao, M. M. , Li, Q. L. , Lin, L. Z. , & Yang, Y. Q. (2017). Preparation of *Moringa oleifera* leaf extracts and their antioxidant activities. Food Science, 39(21), 25–30. 10.7506/spkx1002-6630-201821004

[fsn31015-bib-0028] Zheng, S. Y. , Jin, G. F. , Geng, J. F. , & Yu, L. C. (2015). Comparing nutrients in the seeds of wild soybean and cultivated soybean. Hubei Agricultural Sciences, 54(03), 520–522. 10.14088/j.cnki.issn0439-8114.2015.03.003

[fsn31015-bib-0029] Zheng, Z. J. , Si, D. Y. , Baseer, A. , Li, Z. X. , & Zhang, R. J. (2018). A novel antioxidative peptide derived from chicken blood corpuscle hydrolysate. Food Research International, 106, 410–419. 10.1016/j.foodres.2017.12.078 29579942

[fsn31015-bib-0030] Zhong, Z. S. , Chen, Y. , & Wen, X. L. (2009). Hydrolysis of soybean protein isolate by papain and neutral protease. Modern Food Science and Technology, 25(09), 1039–1042. 10.13982/j.mfst.1673-9078.2009.09.018

